# Maximizing tendency predicts university adjustment and academic performance

**DOI:** 10.3389/fpsyg.2023.1188410

**Published:** 2023-06-07

**Authors:** Mushi Li, Huiyuan Jia, Haixia Wang

**Affiliations:** ^1^School of Psychology and Cognitive Sciences and Beijing Key Laboratory of Behavior and Mental Health, Peking University, Beijing, China; ^2^College of Business Administration, Capital University of Economics and Business, Beijing, China; ^3^School of Journalism & Communication, Jinan University, Guangzhou, Guangdong, China

**Keywords:** maximizing tendency, university adjustment, academic performance, eudaimonic well-being, Grade Point Average (GPA)

## Abstract

**Introduction:**

Decision-making characteristics that contribute to university adjustment and academic performance have been important topics in the research on success in higher education. This study proposes a new perspective that maximizing tendency, as a decision-making style, influences adaptive outcomes in college life.

**Materials and methods:**

Two studies were performed to investigate the positive effects of maximizing tendency on university adjustment and academic performance. In Study 1, we engaged in multistage data collection and surveyed 552 students in four universities. In over a span of 4 years, Study 2 was designed as a time-lagged survey with 309 students.

**Results:**

The results revealed that maximizers among students have better university adjustment after their first year at school and achieve higher GPAs when they finished their bachelor’s degree. Furthermore, eudaimonic well-being mediated the relationship between maximizing tendency and university adjustment (Study 1), whereas university adjustment mediated the relationship between maximizing tendency and college student’s academic performance (Study 2).

**Conclusion:**

These consistent results imply that maximizing tendency as a predictor of university adjustment and academic performance, showing its long-term positive impacts on adaptability and wellbeing.

## 1. Introduction

The maximizing tendency is characterized as a decision-making style that focuses on looking for ideal or optimal choices (Schwartz et al., 2002). It is well recognized that maximizing tendency drives adaptive responses which contribute to life satisfaction (Purvis et al., 2011), graduate students’ job offers and salaries after graduation (Iyengar et al., 2006), and self-efficacy (Rim et al., 2011). However, maximizing tendency was also considered maladaptive (Schwartz et al., 2002), as it was related to worse emotional consequences such as regret, anxiety, and reduced happiness (Schwartz et al., 2002; Purvis et al., 2011; Bruine de Bruin et al., 2016), and exhaustion in the endless search for the maximizing goals (Diab et al., 2008). Built on the trait activation theory (Tett and Burnett, 2003), we attempted to bridge the gap by developing and testing a model of maximizing tendency and its relationships with university adjustment during students’ first year in college and academic performance at the end of the studies.

People with higher maximizing tendency are willing to invest more effort in searching for alternatives while trying to obtain the best outcomes (Dar-Nimrod et al., 2009; Weaver et al., 2015), which reflects two key components for maximization: the maximization goal of choosing the best and the strategy of alternative search (Cheek and Schwartz, 2016). From a motivational perspective, actively pursuing a subjectively high goal leads to a great sense of achievement and satisfaction (Esdar et al., 2016). In other words, maximizers experience more success because of the opportunity to achieve high-level goals (Dalal et al., 2015), and success in turn boosts their happiness (Lyubomirsky et al., 2005). Therefore, those who are successful in academic life are maximizers at work, adapt to new environments more easily, and perform better.

### 1.1. Maximizing tendency and university adjustment

Maximizers always seek for alternative options, and hold high standards (Dalal et al., 2015). Distinguishing between the goals and strategies of maximizers can predict the effective costs they incur. Pursuing the “best” option could be understood in terms of maximizing goals, while an alternative search is a strategy that maximizers may use during decision tasks. Furthermore, recent research has found positive effects of maximizing tendencies, such as eudaimonic happiness. Maximizers may obtain equal or higher levels of happiness compared to satisficers (Diab et al., 2008; Dalal et al., 2015).

Adjustment is the process to fit in a new situation. University adjustment is critical for students’ academic performance and happiness. Good university adjustment normally correlates with academic success, higher grades, and a better chance of success later in life. When young people first enter college, they have to start new peer networks and get used to new academic routines while adapting to the fundamental biological, cognitive, and social changes (e.g., Seidman et al., 2004). Person-environment fit is essential when one’s needs within social contexts change occurs (Jacobs and Eccles, 1993). Young college students are more likely to thrive when social resources they can mobilize to “match” developmental competencies and stage-salient tasks. However, new college students are more likely to experience challenges and risk maladaptive adjustment including psychopathological symptoms where these developmental needs are not matched by the social context (Gutman and Eccles, 2007).

Many students experience major changes in their social environments. Their academic routines, financial responsibilities, and living arrangements are different from high schools. After entering college, many developmental milestones, such as autonomy, and identity development need to be set up for academic success (Arnett, 2016). However, there are also many unexpected challenges for first-year college students. New academic routines can be hard to follow, learning strategies need to be upgraded iteratively, and decision-making responsibilities can be stressful (Larose and Boivin, 1998; Joo et al., 2008). There are many unanticipated psychological discomforts or even symptoms that might present when students cope with challenges (Gall et al., 2000). Many studies reported that students have greater average levels of depression (Cooke et al., 2006; Bewick et al., 2010), stress (Conley and French, 2014), loneliness (Larose and Boivin, 1998), and anxiety (Tao et al., 2000) following the first or second semester of college as compared to the pre-college.

When first entering a new environment, the discomfort always affects one’s wellbeing. After weeks and months of heavy loads in the first semester, stressors in academics, social relationships, and financial concerns resulted in students’ decline in daily feelings and hedonic happiness. A previous study indicated that positive and negative affect represents a high arousal state that may influence students’ emotional trajectories (Rogers et al., 2018). A balance of positive and negative emotional states reflects an individual’s hedonic happiness, but it has little impact on eudaimonic well-being. University is a special new context for first-the year students when they begin their university lives. According to the trait activation theory, the relationship between traits and performance varies depending on the context (Tett and Burnett, 2003). Trait activation theory lays the theoretical foundation for us to establish an explanation of how maximizing tendencies generate adaptive behavior. First, creating demands, known as the expectations about desired behaviors on the part of a group, is the most obvious way that triggers trait activation (Tett and Burnett, 2003). The university campus has a strong atmosphere of competition and actualization. Such context may naturally trigger one’s maximizing tendency, leading them to achieve the acquisition of optimal resources and self-expression by pursuing to be excellent.

Under these circumstances, college students may realize that they must apply the required traits to acquire the necessary resources to accomplish campus goals. Compared with individuals with lower maximizing tendencies, individuals with higher maximizing tendencies have a stronger desire and a more persistent pursuit to achieve a good goal and success (Zhu et al., 2022). Therefore, when the campus environment brings them such a pursuit in an atmosphere of excellence and challenge, college students who want to be the best, will ensure that they can stand out from their peers or achieve the best by actively investing in efforts and setting higher standards for themselves. Driven by such motivation, individuals with higher maximizing tendencies will actively try to adapt themselves to the environment quickly and make positive adjustments to their actions, so that they can have the opportunity to impact the best in any environment.

Besides, maximizing tendency is positively related to one’s achievement motivation (Peng et al., 2018), intrinsic motivation, and self-efficacy (Lai, 2011), which are found to be optimal predictors of one’s adaptability (e.g., Griffin and Hesketh, 2003, 2005; Bell and Kozlowski, 2008; Stokes et al., 2010). From this point of view, the behavioral expression of the maximizing tendency in adaptability will likely be activated during the process of entering college. Hereby, we proposed our first hypothesis:

*H1*: Maximizing tendency is positively related to college students’ adjustment.

### 1.2. The mediating role of eudaimonic well-being

Eudaimonic and hedonic happiness are a pair of concepts well discussed in positive psychology. The concepts specifically address the study of happiness, quality of life, and resources (Csikszentmihalyi and Seligman, 2000). A definition of eudaimonic well-being describes meaningful happiness in twofold: self-actualization and personal growth at the individual level (Ryff, 1989), and achieving environmentally shared goals and values at the social level (Massimini and Delle Fave, 2000).

Nevertheless, the pursuit of the meaning of life, the realization of life goals, and the maintenance of important relationships characterizes human beings as cultural animals (Baumeister, 2005). These characteristics were described in eudaimonic theories and constructs, such as psychological happiness (Ryff, 1989), personal expressiveness (Waterman et al., 2008), sense of coherence (Antonovsky, 1987), self-determination (Ryan and Deci, 2001), and psychological selection (Csikszentmihalyi and Massimini, 1985). Studies have investigated the relationship of meaning with happiness, purpose in life, goal pursuit and achievement, and development of personal potential (Morgan and Farsides, 2009).

In daily life, college students encounter worries about adaptation to the new environment, academic performance, workload, future employment, etc. However, adverse life circumstances foster personal growth (Ardelt et al., 2010). Besides, whether or not an individual can adequately deal with these stressors and achieve good feelings of well-being, depends to a large extent on the individual’s ability to face them in an adaptive manner (Skinner et al., 2013). In other words, achieving eudaimonic well-being often happens through goal attainment or personal growth, resulting in one’s university adjustment.

“Eudaimonic aspirations” focus on seeking meaning and expressing important values (McGregor and Little, 1998). Compared to hedonic happiness produced by daily life and emotional change, eudaimonic well-being may last longer and have a deeper influence on one’s adjustment. Research has found that maximizers experience more eudaimonic well-being compared to satisficers (Rim et al., 2011; Oishi et al., 2014; Kokkoris, 2016). Maximizing decision-making style is domain-specific and varies within a person. Maximizers among students usually value their academic success heavily, so they may put more effort into their studies. Meanwhile, getting used to the new academic stage and learning new coping strategies in the first year of college is vital in students’ adjustment phase. We assume student maximizers may adapt to new college life by obtaining more eudaimonic well-being; hence, we propose our hypothesis 2:

*H2*: Eudaimonic well-being mediates the relationship between maximizing tendency and college students’ adjustment.

### 1.3. Maximizing tendency and academic performance

Maximizers are more willing to establish a high-level goal of optimal choice, thereby expecting an elusive outcome (Iyengar et al., 2006; Weinhardt et al., 2012; Giacopelli et al., 2013; Benjamin et al., 2014). When possessing a high standard of academic goal, one may expend additional effort to seek potential options (Dar-Nimrod et al., 2009). The prediction and explanation of academic performance and the investigation of the factors relating to academic success and persistence in students are topics of utmost importance in higher education (Ruban and McCoach, 2005).

Maximizers will constantly accumulate a sense of autonomy brought by participation and devotion in the process of constantly achieving goals. Keeping high standards in academics encourages them to persevere and foster strong motivation to achieve goals, which are beneficial for their academic performance. Therefore, we proposed our hypothesis 3:

*H3*: Maximizing tendency is positively related to college student’s academic performance.

Academic performance is an objective reflection of the results of college students’ adjustment. Maximizers have high motivation to succeed in the academic domain. This study investigated that maximizing tendency, especially the goal setting and goal-attaining process, reflected students’ behavior to coordinate the pursuit of academic goals and their adaptation to the new college environment. This research strongly echoes the tenet of trait activation theory (Tett and Burnett, 2003), which states that college students choose to engage in more adaptive behaviors because contextual cues require them to express themselves to get the resources they need. In a longer period, maximizers who are better able to adapt to college life will improve their academic performance accordingly. Thus, we proposed our hypothesis 4:

*H4*: College students’ adjustment mediates the relationship between maximizing tendency and their academic performance.

**Figure 1:**
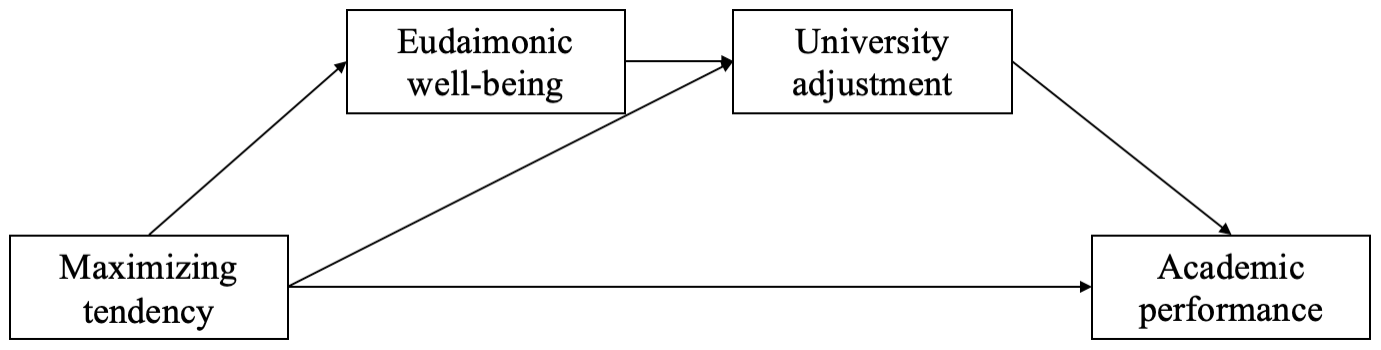
Research model.

In the present research, based on the trait activation theory (Tett and Burnett, 2003), we proposed a research model to test maximizing tendency and its relationships with university adjustment and academic performance (Figure 1). Two studies were conducted to test the hypothesis. Study 1 was conducted with first-year students during their first year of university. Students were asked to complete three surveys at the first month of their entry to the university, the end of the first semester, and the end of the second semester (i.e., the end of the first school year), respectively. This study tested the effect of maximizing tendency on university adjustment, and the mediating role of eudaimonic well-being. Study 2 lasted 4 years. Students were required to complete two surveys at the first semester of the university and at the end of the first school year, respectively. The final GPA (academic performance) was collected after their graduation (4 years later). This study examined the influence of maximizing tendency on academic performance, and the mediating role of university adjustment. Meanwhile, it replicated the relationship between maximizing tendency and university adjustment.

## 2. Study 1: maximizing tendency and university adjustment

### 2.1. Sample and procedure

Participants were first-year students from four universities in China. Data were collected in three phases using paper-and-pencil surveys with an interval of one semester to minimize potential common method bias and to reduce respondent fatigue (Podsakoff et al., 2003). Surveys were distributed to these first-year students through their teachers in the class. At Time 1 (the first month of first-year students entering college), we collected data on maximizing tendency and demographics (age, and gender). Time 2 survey was measured at the end of the first semester. Students were asked to report their eudaimonic well-being. At Time 3, we collected data on the adjustment (students self-reported) at the end of the first school year.

Seven hundred and twenty-nine first-year students enrolled in this research. Because of missing data and voluntary withdrawal, we had a final sample of 552 students (response rate 75.5%), of which 376 were women (68.1%). The average age was 18.37 years (SD = 1.12). The distribution of participants in universities was 25.9% (University A), 12.1% (University B), 28.1% (University C), and 26.1% (University D). These four universities were located in four different cities. Students were from different majors, including psychology, management, sciences, and languages.

### 2.2. Measures

#### 2.2.1. Maximization tendency

Maximization tendency was measured at Time 1 (the beginning of the first school year), using a 9-item scale called Maximizing Tendency Scale (Diab et al., 2008). The scale measured two components of maximization, satisficing, and alternative search. Participants were asked to rate the extent to which they agreed or disagreed with statements such as “No matter what it takes, I always try to choose the best thing.” on a 7-point scale (from 1 = strongly disagree to 7 = strongly agree). Cronbach’s alpha coefficient for maximization tendency was 0.78.

#### 2.2.2. Eudaimonic well-being

Eudaimonic well-being was measured at Time 2 (the end of the first semester), using a 21-item scale developed by Waterman et al. (2010). Participants were asked to rate the extent to which they agree or disagree with statements on a 5-point scale (1 = strongly disagree to 5 = strongly agree). An item sample is: “I believe I have discorded who I really am.” Cronbach’s alpha coefficient for eudaimonic well-being was 0.94.

#### 2.2.3. University adjustment

University adjustment was measured at Time 3 (the end of the first school year) by using the sixty-item university adjustment scale developed by Fang et al. (2005). Participants were asked to rate the extent to which they agree with the descriptions of their college lives in the last 1 month on a 7-point scale (1 = not agree at all, 5 = totally agree). Item samples are: “I like my major very much”; “I quite feel comfortable in my dormitory”; and “I am always energetic.” Cronbach’s alpha coefficient for adjustment was 0.93.

#### 2.2.4. Control variables

Control variables of gender and age were included in this study to reduce spurious results owing to the potential influence of demographic characteristics.

### 2.3. Results

The means, standard deviations, and zero-order correlations of key variables are shown in Table 1. Since age and gender were not significantly related to eudaimonic well-being and university adjustment, they were excluded from the further analysis.

**Table 1:** Descriptive statistics and correlations.

	Variables	Mean	SD	1	2	3	4	5
1	Age	18.37	1.12	–				
2	Gender	1.68	0.47	−0.01				
3	Maximizing tendency (T1)	4.52	1.14	−0.05	0.01			
4	Eudemonic well-being (T2)	5.24	0.81	0.02	−0.02	0.20**		
5	University adjustment (T3)	3.47	0.45	0.11*	−0.06	0.11*	0.13**	–

*N* = 552. Gender: 1-male, 2-female. T1–T3 = Time 1 to Time 3. *p > 0.05, **p < 0.01, two-tailed test.

We found that maximizing tendency positively predicted university adjustment (*β* = 0.11, *t* = 2.24, *p* = 0.016); therefore, Hypothesis 1 was supported. Additionally, maximizing tendency positively predicted eudaimonic well-being (*β* = 0.20, *t* = 4.74, *p* < 0.001), which is consistent with the previous research findings.

Hypothesis 2 predicted that eudaimonic well-being acted as the mediator in the relationship between maximizing tendency and adjustment. We utilized Model 4 in PROCESS macro (Preacher and Hayes, 2004; Hayes, 2013) to test our hypotheses. Specifically, the maximizing tendency was the independent variable and eudaimonic well-being was the mediator in the model. The independent variable, mediator, and control variables predicted the dependent variable (i.e., adjustment). The results of the regression analyses are shown in Table 2. In addition, we also ran the mediation model without including the control variables, and the results remained consistent in both cases.

**Table 2:** Mediation regression analyses predicting eudaimonic well-being and university adjustment.

	First stage dependent variable = Eudaimonic well-being			Second stage dependent variable = University adjustment		
	*Β*	*SE*	*t*	*β*	*SE*	*t*
*Control variable*						
Age	0.02	0.03	0.64	0.04	0.02	2.46*
*Independent variable*						
Maximizing tendency	0.14	0.03	4.22**	0.03	0.02	1.42
Eudaimonic well-being				0.08	0.03	3.18*
*F*	9.08***			6.97***		
*R^2^*	0.04			0.05		

*N* = 552. *p > 0.05, **p < 0.01, ***p <0.001, two-tailed test.

We adopted 5,000-times resampling-based bootstrapping methods and examined the 95% bias-corrected bootstrapped confidence interval 95% C.I [0.0018, 0.0275]. Since these confidence intervals did not include zero, Hypothesis 2 was supported.

## 3. Study 2: maximizing tendency and academic performance

### 3.1. Sample and procedure

Participants were first-year students from two universities in China. Data were collected in three phases. At Time 1 (in the first month of first-year students entering college), the questionnaires were distributed through their teachers in the class, measuring their self-efficacy, maximizing tendency, and demographic data (age, and gender). Time 2 survey was measured at the end of the first semester. Students were asked to report their adjustments. Time 3 was measured 4 years later (after graduation). With students’ permission, the university provided their Grade Point Average (GPA). Five hundred first-year students enrolled in this research; because of voluntary withdrawal, a final sample of 309 students (response rate 61.8%) remained, of which 232 were females (75.1%). The average age was 18.19 years (*SD* = 0.82).

### 3.2. Measures

#### 3.2.1. Maximization tendency

Maximization tendency was measured at Time 1 (the beginning of the first school year), using a different scale from that of Study 1. The scale used in Study 2 was Maximization Scale (Nenkov et al., 2008) with 13 items. The scale measured three components of maximization, alternative search (e.g., I often fantasize about living in ways that are quite different from my actual life), decision difficulty (e.g., When shopping, I have a hard time finding clothing that I really love), and high standards (e.g., No matter what I do, I have the highest standards for myself). Participants were asked to rate the extent to which they agreed or disagreed with statements on a 7-point scale (from 1 = strongly disagree to 7 = strongly agree). Cronbach’s alpha coefficient for maximization tendency was 0.67.

#### 3.2.2. University adjustment

The adjustment was measured at Time 3 (the end of the first school year) using the same scale used in Study 1. Cronbach’s alpha coefficient for adjustment was 0.82.

#### 3.2.3. Academic performance

Academic performance was evaluated by student’s GPA (in four years). Each grade was assigned with numerical values, which are course grade points. They are multiplied by the number of course credits. The GPA is the sum of four years’ resulting production, and then divided by the total number of course credits.


$$\mathrm{GPA}=\frac{\Sigma \left(\mathrm{Course Grade Point}\times \mathrm{Course Credit}\right)}{\Sigma \;\mathrm{Course Credit}}$$

#### 3.2.4. Control variables

Control variables of gender, age, and self-efficacy were included in this study to reduce spurious results owing to the potential influence of personal characteristics.

### 3.3. Results

The means, standard deviations, and zero-order correlations of key variables are shown in Table 3. Age, gender, and self-efficacy were not significantly related to college students’ adjustment and GPA; and were hence excluded from the further analysis.

**Table 3:** Descriptive statistics and correlations.

Variables		Mean	SD	1	2	3	4	5	6
1	Age	18.19	0.82	–					
2	Gender	0.75	0.43	0.02					
3	Self-efficacy	3.46	0.50	0.04	−0.09				
4	Maximizing tendency (T1)	4.36	0.76	−0.07	0.123*	0.02			
5	University adjustments (T2)	2.95	0.82	−0.06	0.04	−0.06	0.160**		
6	GPA (T3)	3.43	0.47	−0.11	0.28	−0.09	0.169**	0.284**	–

*N* = 309. Gender: 0-men, 1-women; T1–T3 = Time 1 to Time 3; *p > 0.05, **p < 0.01, two-tailed test.

Our findings indicated that maximizing tendency positively predicted GPA (*β* = 0.17, *t* = 2.94, *p* = 0.004), therefore supporting Hypothesis 3. Additionally, maximizing tendency positively predicted college students’ adjustment (*β* = 0.16, *t* = 2.71, *p* = 0.007), replicating the results found in Study 1. Hence, University adjustment was positively related to GPA (*β* = 0.28, *t* = 5.06, *p* < 0.001).

We further adopted 5,000-times resampling-based bootstrapping methods and examined the 95% bias-corrected bootstrapped confidence interval 95% C.I [0.0052, 0.0535]. As these confidence intervals did not include zero, Hypothesis 4 was supported, showing that college students’ adjustment mediates the relationship between their maximizing tendency and academic performance (Table 4).

**Table 4:** Mediation regression analyses predicting adjustment and GPA.

	First stage dependent variable = College students’ adjustment (T2)			Second stage dependent variable = GPA (T3)		
	*β*	*SE*	*t*	*β*	*SE*	*t*
Maximizing tendency	0.17	0.06	2.71**	0.08	0.04	2.29*
College students’ adaptation				0.15	0.03	4.41***
*F*	7.35**			14.33***		
*R^2^*	0.03			0.09		

*N* = 309. *p > 0.05, **p < 0.01, ***p < 0.01 two-tailed test.

## 4. Discussion

New students in college may encounter major life changes (Pancer et al., 2000), many of which need positive adjustments that are demanding and can create significant coping challenges for incoming college students (Conley and French, 2014). This research found that maximizing tendency as a decision-making style positively predicted college students’ adjustment and long-term academic performance. By taking two longitudinal studies, it was confirmed that maximizing tendency has a clear predictive effect on the individual’s adjustment and performance in the long run. Also, eudaimonic well-being played an important role in explaining this relationship. Noteworthily, this study tracked the fourth year of academic performance to reveal the relationship between maximizing tendency, college student’s adjustment, and their final evaluation of academic success. Maximizing tendency positively predicted college students’ adjustment and their GPA, and this effect was stable across two studies. Besides, eudaimonic well-being acted as the mediator in the relationship between maximizing tendency and adjustment in Study 1. College students’ adjustment mediated the relationship between maximizing tendency and academic performance in Study 2. These consistent results imply that maximizing decision-making style could be a new perspective to understand students’ adjustment and academic performance.

Maximizers are described as those who “desire the best possible result,” compared to satisficers who “desire a result that is good enough to meet some criterion” (Schwartz et al., 2002). Maximizers are less satisfied with the outcomes of their decisions and may experience more negative emotions (e.g., Schwartz et al., 2002; Iyengar et al., 2006; Polman, 2010; Leach and Patall, 2013; Shiner, 2015). However, maximizers have been found to have a positive correlation with in-role performance (Giacopelli et al., 2013), more job offers, better job opportunities, and higher salaries after graduation owing to these characteristics (Iyengar et al., 2006).

Our study is the first to explore the link between students’ maximizing tendency, university adjustment, and academic performance; however, a noteworthy question is: Is it possible to obtain better academic results by adopting the maximizing decision-making strategy? The answer would be “yes,” since maximizers have proven that determination of their long-term efforts could bring them better results. However, this can be costly as they also tend to experience increased emotional outbreaks, such as regret, sadness, or even depression, momentarily.

Academic success is arguably the most important part of a student’s college life, and hence they are most focused on it. After all, academic performance is critical in applying for graduate school, finding a good job, getting a decent salary, or even for elevated status. Correctly evaluating subjective task value before decision-making directly affects one’s motivation, choice, effort investment, and performance (Eccles, 2005). Motivation increases when the subjective value of a task is high (Eccles and Wigfield, 2002). Having a strong motivation for university adjustment and academic success, maximizers are more willing to persist and pursue higher goals, thus leading to more investment in effort.

Based on tracing the relationship between maximizing tendency and well-being, this study introduces the realization of well-being as a supplement to traditional subjective well-being and expands the framework of well-being research. At the same time, we refute the negative argument of the maximizing paradox by understanding the process of maximizing individuals seeking goals and challenging goals from the observational perspective of two types of well-being. It has been pointed out that the optimal individual has higher realization motivation and realization-related well-being.

When we view eudaimonic well-being as a positive psychological resource, the process of adaptation can be thought of as a behavioral pattern for acquiring and maintaining resources (Gawke et al., 2017). College students choose more adaptive behaviors because contextual cues require them to express themselves to get the resources they need. The pursuit of eudaimonic well-being is more obvious in maximizers; individuals who always strive to choose the best alternative may experience an increased pleasure deriving from the fulfillment of their potential and a sense of authentic, meaningful, and self-expressive living (Kokkoris, 2016).

Combined with trait activation theory and broaden-and-build-theory, maximizing tendency in the college transition phase may help students focus on their major goal, which is academic success. They may suffer from momentary emotional challenges or roadblocks in their major courses during the entire semester. However, eudaimonic well-being would pay them back if they got a few ‘A’ scores on their quizzes or midterms, and this pattern would last and then form an upward spiral. In this upward spiral, eudaimonic well-being continuously fuels their motivation to succeed. Maximizers are hence encouraged to advance gradually, immersing themselves in the new environment, and finally getting a better university adjustment and academic performance.

The theoretical contributions of our research are mainly reflected in the following aspects. First, we demonstrated that the maximizing decision-making style has a long-term positive effect on college students’ adjustment and academic performance by providing continuous eudaimonic well-being. Second, students have high motivation to adopt maximizing strategies in the academic domain because they assign high value to it. The maximizing tendency in college students has brought them better academic performance, and led to a higher level of eudaimonic well-being. This result could help researchers to understand and explore maximizing strategies from the perspective of cognitive evaluation (Zhu et al., 2022).

The practical contributions of this research could be explained in two parts. First, maximization as a decision-making style is worth cultivating consciously. A good student is always expecting a good grade. Academic success could be extremely helpful for one’s learning motivation or confidence, even future choices at different life stages. Though existing literature fully considered motivation, self-efficacy, attribution, goal orientations, etc., (Linnenbrink and Pintrich, 2002), maximizing tendency as a decision-making style has not been given due attention.

Second, maximizers are more likely to experience negative affect and anxiety as a result (Patalano et al., 2015). Although eudaimonic well-being is constant in maximizers, their hedonic happiness may fluctuate. Being mentally unstable could be a risk factor for one’s well-being and performance in life (Eisenberg et al., 2002). Relevant departments or facilities of college should pay attention to the mental health status of maximizers.

The current study had some limitations that could be addressed in future works. First, most variables were measured via self-report, which may not completely avoid the common method bias. Future research could also collect a broader range of experiences, especially changing feelings and emotions of students when they first enter college. In addition, for academic performance, various methods other than GPA, such as personal evaluation or school evaluation may also be considered. Taken together, the long-term positive effect of maximizing tendency on university adjustment and academic success could be more robust. Third, maximizing tendency was measured at the beginning of student’s university life. Although maximization is concerned as a trait, but it is still possible to be changed in 4 years. Future research may concern the dynamic change of maximizing tendency in a long-term perspective.

We have all experienced the turmoil around the course selection week. The adaptation of students may be significant in the first month or even the first 2 weeks, and many special circumstances are encountered during this period. Collecting time slots in current data is based on semesters; setting more time nodes by month may bring us more information about students’ adjustments.

Because of the length of the questionnaire, we did not collect data on hedonic happiness and momentary feelings such as anxiety or depressive mood. From our results, it was evident that these transient emotions did not affect the results of maximizers’ adaptation to their college life. However, in the adjustment process, maximizers may experience stronger mood swings than satisficers, and put more effort to stick to their goals, promoting greater self-control and self-actualization.

Future research can further explore the internal psychological mechanism of maximizing the tendency of university adjustment and academic performance. First, the personality components represented by maximizing tendencies may form definite stress-coping styles, which is the basis for the hypothesis of this study. However, in this study, we did not measure the coping styles; hence it is doubtful whether the coping style underlies the process from maximizing tendency to adaptation. If maximizing tendencies do behave as we hypothesize on behavioral strategies, then high standards of positive traits in the maximizing component should correlate more with positive coping performance, and decision difficulties should correlate more with negative coping styles. The addition of this link helps fill in the process chain from maximizing tendencies to adaptation outcomes and gain a more comprehensive understanding of the process from personality to well-being outcomes.

The application of maximizing tendency and its related concepts in the field of college education could be further expanded. In maximization decision-making style research, maximizing tendency had better behavioral consequences on the job-seeking behavior and job-seeking results of college students (Iyengar et al., 2006). In college life, students actively seek and manage their short-term and long-term goals, and adjust the goal in this process. From the perspective of goal motivation and personal well-being, this process is an excellent starting point for exploring the long-term effects of maximizing tendencies. The degree of fit between the concept of maximizing tendency itself and the group of college students, and its controversial conclusions on subjective well-being will enlighten college education: whether the pursuit of the best goal is worth advocating and whether the difference in maximizing tendency will lead to differences in students’ achievement and success in college life and even future career. Answers to these questions and further exploration will be helpful to determine the positive effect of maximizing tendency on the achievement of personal goals. Further, relevant psychological variables, such as college students’ mental health, perfectionism, anxiety, and depression, need to be carefully considered when utilizing maximizing tendency as a positive personal resource.

## 5. Conclusion

Research indicates that maximizing tendency has positive impacts on job performance (Giacopelli et al., 2013) and eudaimonic wellbeing (Kokkoris, 2016). However limited evidence indicates that the adaptability of maximizing tendency. Our research adds to this body of research, extending the positive consequences of maximizing tendency. The results showed that maximizing tendency plays a major role in predicting university adjustment and academic achievement. Given this evidence, it appears that scholars may underestimate the adaptability of maximizing tendency. Rather, investigating the role of maximizing tendency in predicting positive behaviors and outcomes represents a promising untapped domain for future research.

## Data availability statement

The raw data supporting the conclusions of this article will be made available by the authors, without undue reservation.

## Ethics statement

The studies involving human participants were reviewed and approved by the Ethics Committee of College of Business Administration, Capital University of Economics and Business. The patients/participants provided their written informed consent to participate in this study.

## Author contributions

ML was responsible for writing and data analysis. HJ was responsible for data collection, data analysis, and conceptualization. HW was responsible for editing and supervising. All authors contributed to the article and approved the submitted version.

## Funding

This research was financially funded by National Natural Science Foundation of China (72002139 and 72174075), and R&D Program of Beijing Municipal Education Commission (SM202210038015).

## Conflict of interest

The authors declare that the research was conducted in the absence of any commercial or financial relationships that could be construed as a potential conflict of interest.

## Publisher’s note

All claims expressed in this article are solely those of the authors and do not necessarily represent those of their affiliated organizations, or those of the publisher, the editors and the reviewers. Any product that may be evaluated in this article, or claim that may be made by its manufacturer, is not guaranteed or endorsed by the publisher.
